# Africa and the global carbon cycle

**DOI:** 10.1186/1750-0680-2-3

**Published:** 2007-03-07

**Authors:** Christopher A Williams, Niall P Hanan, Jason C Neff, Robert J Scholes, Joseph A Berry, A Scott Denning, David F Baker

**Affiliations:** 1 Natural Resource Ecology Laboratory, Colorado State University, Fort Collins, CO 80523, USA; 2 University of Colorado, Boulder, CO 80309, USA; 3 Council for Scientific and Industrial Research, Pretoria 001, South Africa; 4 Carnegie Institution of Washington, Stanford, CA, 94305, USA; 5 Department of Atmospheric Sciences, Colorado State University, Fort Collins, CO 80523, USA; 6 National Center for Atmospheric Research, Terrestrial Science Section, Climate and Global Dynamics Division, 1850 Table Mesa Dr., Boulder, CO 80307, USA

## Abstract

The African continent has a large and growing role in the global carbon cycle, with potentially important climate change implications. However, the sparse observation network in and around the African continent means that Africa is one of the weakest links in our understanding of the global carbon cycle. Here, we combine data from regional and global inventories as well as forward and inverse model analyses to appraise what is known about Africa's continental-scale carbon dynamics. With low fossil emissions and productivity that largely compensates respiration, land conversion is Africa's primary net carbon release, much of it through burning of forests. Savanna fire emissions, though large, represent a short-term source that is offset by ensuing regrowth. While current data suggest a near zero decadal-scale carbon balance, interannual climate fluctuations (especially drought) induce sizeable variability in net ecosystem productivity and savanna fire emissions such that Africa is a major source of interannual variability in global atmospheric CO_2_. Considering the continent's sizeable carbon stocks, their seemingly high vulnerability to anticipated climate and land use change, as well as growing populations and industrialization, Africa's carbon emissions and their interannual variability are likely to undergo substantial increases through the 21st century.

## Background

Africa stands out among continents for widespread and deeply entrenched poverty, slow economic development, and agricultural systems prone to failure during frequent and persistent droughts [1]. Africa is also home to some rapidly developing economies, tremendous natural resources and remarkable social and ecological diversity. The unique history of Africa, the close dependencies of people on natural resources and a future that will certainly include substantial industrial, agricultural and social development, suggest that Africa will become a key player in the carbon cycle of the 21st century. However, our knowledge about Africa's current role in the global carbon cycle remains remarkably limited. We currently do not know whether Africa is a net sink or source of atmospheric carbon, and have only vague indications of the continent's temporal and spatial patterns of carbon exchange. Given the current development agenda that is intended to elevate Africa's importance in the global economy [2], it is time to focus as well on Africa's role in the global carbon cycle. Here we review what is known about Africa's carbon dynamics from regional and global inventories, and forward and inverse model analyses, and highlight some of the unique features of Africa's contribution to global carbon fluxes.

The diverse elements of the global carbon cycle have been the focus of much recent research [3-5]; research that is vital to our understanding of the missing carbon sink, future atmospheric carbon dioxide concentrations, and future climate [6-8]. Much of that research has concentrated on carbon dynamics of the large ocean basins [9,10] and terrestrial exchange in North America [11,12] and Eurasia [13,14]. Despite representing 20% of the global land mass, Africa has thus far been largely neglected in these studies. Africa contributes a disproportionately small fraction of the global fossil fuel carbon emissions that are responsible for rising atmospheric carbon dioxide concentrations, with 14% of global population [15], but only 3% of fossil emissions [16]. In contrast, Africa plays a globally important role in fire and land use carbon emissions, though the magnitudes of these terms are highly uncertain.

To date, continental assessments of Africa's carbon dynamics are primarily model-based. Plausible estimates of Africa's regional sources and sinks can potentially be supplied by atmospheric inversion using global CO_2 _concentration measurements and atmospheric transport models. However such 'top-down' solutions have large uncertainties, particularly for Africa and other tropical regions, due to the paucity of appropriately located CO_2 _concentration measurements [17,18]. Existing data are also insufficient to partition carbon sources and sinks within Africa, and inversion techniques provide little insight regarding mechanisms responsible for net uptake and release of carbon in space and time [17,19,20]. The alternative approach is to perform spatially, temporally and source-differentiated 'bottom-up' estimation using biogeochemical models. However, such regional carbon flux estimates are only weakly supported by the sparse network of place-based observations, and thus are largely founded on models that have been parameterized and evaluated with extra-African observations. The resultant uncertainty reduces our ability to resolve African and global carbon sources and sinks, and hinders wise resource management in Africa for greenhouse gas mitigation.

With the hope of identifying carbon cycle research priorities that may be met through focused research efforts, we synthesize current understanding of carbon stocks and fluxes within Africa, highlight uncertainties in those terms, and diagnose where uncertainty in our knowledge of the African carbon cycle impacts our ability to assess global carbon dynamics. We then outline where new measurements and further research are most likely to contribute understanding of African and global carbon cycling, and discuss implications for African involvement in international climate change agreements.

## Africa in the Balance

**Figure 1:**
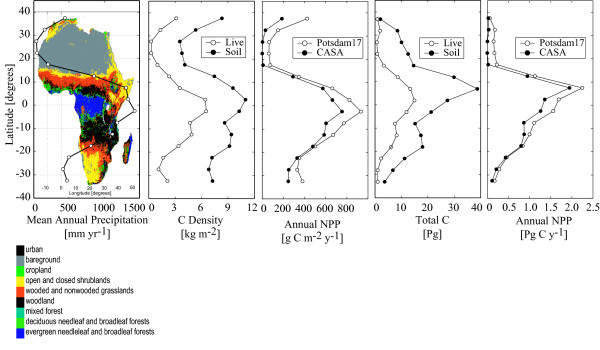
Latitudinal distribution of mean annual precipitation [72], soil [78] and plant [84] carbon density, annual net primary production (NPP) per unit ground area from CASA [23, 26] and the Potsdam 17-model intercomparison [89], total soil and live carbon, total annual NPP, shown with the spatial distribution of land cover [106] (colors). Means and totals were calculated from published data using all terrestrial locations in 5° latitude zones.

Africa is second only to Eurasia in continental surface area. It has large areas of moist tropical forest, seasonal and semi-arid woodland, savanna, grassland and desert, as well as smaller regions of Mediterranean and montane vegetation in extra-tropical and high elevation areas (Figure 1).

**Figure 2:**
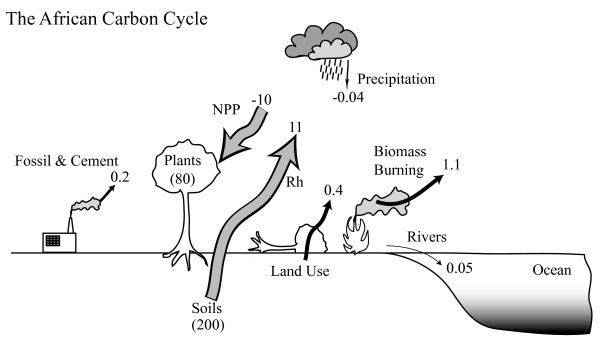
The African carbon cycle. Annual fluxes and pools (shown in parentheses) all in units of 10^15 ^g C, where NPP is net primary production, and Rh is heterotrophic respiration. Estimates as reported in Table 1

Initial estimates [21] of carbon stocks and the various flux pathways (Table 1, Figure 2) suggest that the continent plays a significant role in atmospheric CO_2 _dynamics at time scales ranging from sub-seasonal to decadal and longer. The balance of terms in Figure 2 should not be interpreted as identifying a large net biotic source for the continent but rather that independent studies which estimate the magnitude of fluxes associated with individual pathways can not be used in a budget calculation without careful consideration of the processes represented in each estimate and the associated uncertainties. For example, biomass burning emissions are not modeled explicitly in many biogeochemical or biophysical models and may thus be effectively lumped into heterotrophic respiration.

**Table 1:** Global terrestrial and African carbon stocks and fluxes representative of the 1990s.

	**Global Total**	**Africa Total**	**Africa/Global**	**Global Citations**	**Africa Citations**
**Land Area [10**^12^**m**^2^**]**	**148.8**	**30.2**	**0.20**	--	--
**Human Population [10**^9^**]**	**6.38**	**0.87**	**0.14**	[15]	[15]
**Soil Carbon**	**1600 ± 220 (2060, 1395)**	**200 ± 50 (240, 170)**	**0.13**	[77–81]	[22, 81]
**Live Plant Carbon**	**610 ± 47 (654, 559)**	**80 ± 28 (105, 50)**	**0.13**	[77, 80, 82, 83]	[22, 84, 85]
**Net Primary Production**	**-56 ± 10 (-72, -37)**	**-10 ± 3 (-13, -7)**	**0.19**	[24-26, 80, 83, 86-89]	[22, 24–26, 89]
**Heterotrophic Respiration**	**57 ± 17 (81, 34)**	**11 ± 5 (-18, -7)**	**0.19**	[24, 87, 90, 91]	[24, 91]
**Fossil Emissions**	**6.2 ± 0.2 (6.4, 6.0)**	**0.2 (--)**	**0.03**	[16, 77, 92]	[16]
**Net Land Use Emissions**	**1.7 ± 0.71 (2.8, 0.8)**	**0.36 ± 0.05 (0.4, 0.3)**	**0.21**	[32, 77, 92, 93]	[31, 32, 94]
Deforestation	1.42 ± 0.64 (2.2, 0.5)	0.24 ± 0.12 (0.37, 0.08)	0.17	[29, 32, 33, 95, 96]	[32, 33]
Conversion to Crops	0.83 ± 0.17 (1.0, 0.6)	0.10 ± 0.01 (0.11, 0.08)	0.12	[24]	[24, 31]
**Biomass Burning**	**2.9 ± 0.9 (4.7, 1.5)**	**1.1 ± 0.5 (1.8, 0.3)**	**0.37**	[27, 28, 40, 76, 97–100]	[40, 76, 98, 100, 101]
Deforestation	0.36 ± 0.26 (0.89, 0.13)	0.07 (--)	0.19	[27, 76, 97–100]	[76]
Shifting Cultivation	0.60 ± 0.30 (1.13, 0.34)	0.24 (--)	0.41	[27, 28, 76, 97–99]	[76]
Savanna Fires	1.45 ± 1.14 (4.1, 0.22)	1.47 ± 0.33 (1.67, 1.09)	1.02	[27, 28, 41, 76, 97–99]	[41, 76, 98, 102]
Fuel wood	0.51 ± 0.36 (1.26, 0.28)	0.16 ± 0.08 (0.24, 0.08)	0.32	[27, 28, 76, 97–99]	[76]
Agricultural Residues	0.41 ± 0.24 (0.80, 0.13)	0.01 (--)	0.03	[27, 28, 76, 97–99]	[76]
**Riverine C Discharge**	**0.71 ± 0.13 (0.8, 0.62)**	**0.055 ± 0.021 (0.07, 0.04)**	**0.08**	[35, 70]	This study
DIC	0.41 ± 0.03 (0.44, 0.38)	0.040 ± 0.014 (0.05, 0.03)	0.10	[35, 70]	This study
DOC	0.29 ± 0.16 (0.40, 0.18)	0.017 ± 0.006 (0.021, 0.013)	0.06	[35, 70]	This study
**Precipitation C Flux**	**-0.51 ± 0.17 (0.68, 0.34)**	**-0.036 ± 0.025 (-0.054, -0.018)**	**0.07**	[71]	This study
DIC	-0.08 ± 0.02 (-0.10, -0.06)	-0.009 ± 0.006 (-0.013, -0.004)	0.11	[71]	This study
DOC	-0.43 ± 0.15 (-0.58, -0.28)	-0.028 ± 0.019 (-0.041, -0.014)	0.06	[71]	This study
**CH**_4_**Emissions**	**0.33 ± 0.11 (0.40, 0.25)**	**0.007 (--)**	**0.02**	[77, 103]	This study
CH_4 _from fires	0.02 ± 0.01 (0.03, 0.01)	0.005 ± 0.001 (0.007, 0.004)	0.33	[40, 41]	[40, 41]
**CO Emissions**	**0.30 ± 0.09 (0.36, 0.24)**	**0.07 (--)**	**0.24**	[77, 105]	[105]
CO from fires	0.27 ± 0.14 (0.51, 0.12)	0.09 ± 0.02 (0.11, 0.07)	0.32	[40] [27, 28]	[40]
**NMHC Emissions**	**0.15 (--)**	**0.05 ± 0.04 (0.08, 0.02)**	**0.33**	[105]	[46, 105]
**Net Biomass Trade**	**0.023 (--)**	**0.038 (--)**	**NA**	[47]	[47]
Gross Import	-0.345 (--)	-0.023 (--)	0.07	[47]	[47]
Gross Export	0.370 (--)	0.061 (--)	0.16	[47]	[47]

NA is not applicable, DIC is dissolved inorganic carbon, DOC is dissolved organic carbon, NMHC is non-methane hydrocarbon, biomass import and export are the sum of cereal, paper, and wood products.

Shown are means and standard deviations of published estimates with maximum and minimum values in parentheses. Positive fluxes refer to a terrestrial source from Africa to the atmosphere, ocean, or other land masses as appropriate. Carbon stocks and fluxes have units of Pg C and Pg C y^-1^, respectively. See Appendices 1 for methods of calculation. Inconsistencies among tabular values arise in part due to inclusion of studies that report only some of the terms and between study variation in methods of estimation (e.g. through use of reports containing global but not African deforestation rates).

Patterns of soil and vegetation carbon stocks and net primary production (NPP) are highly correlated with annual rainfall (Figure 1). Africa's fraction of global annual NPP is estimated to be similar to the fractional terrestrial area of the continent (Table 1 and Figure 1); the large unproductive arid regions are compensated by high productivity in forests and woodlands. Carbon stocks and NPP per unit land area center on the equator and decline to the north and south toward increasingly arid environments. However, greater land area in Africa's northern hemisphere cause latitudinally summed C stocks and NPP to peak north of the equator (Figure 1).

African fossil fuel emissions are a tiny fraction of global totals, even when normalized by land area or human population (Table 1), while renewable energy sources (wood, charcoal) are a substantial component of domestic emissions. With low fossil emissions, Africa's current continental scale carbon fluxes are dominated by biogenic uptake and release from terrestrial ecosystems as well as pyrogenic emissions in savanna and forest fires. As is generally true globally, the continent's large carbon uptake from photosynthesis is offset by an equivalently large respiration flux, leading to near-zero net biotic flux at multi-year or longer timescales. In spite of these broad patterns, estimates can differ widely between studies (Table 1) and temporal variability is large.

**Figure 3:**
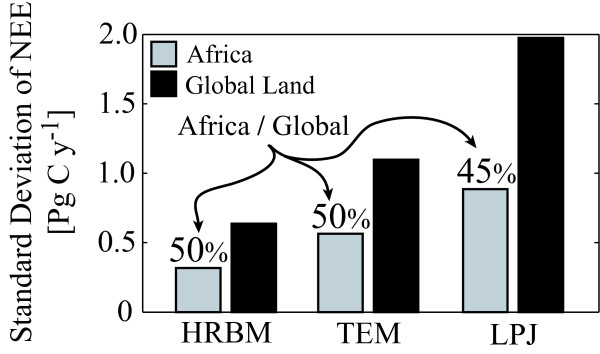
Standard deviation of net ecosystem carbon exchange (NEE) estimated with three ecosystem models, High Resolution Biosphere Model (HRBM), Terrestrial Ecosystem Model (TEM), and Lund-Potsdam-Jena model (LPJ) as reported by McGuire et al. [24].

Bottom-up simulation models [22-24] indicate large interannual variation in Africa's net ecosystem carbon exchange (NEE), with an interannual variability (expressed as the standard deviation of annual NPP) that is approximately 50% of the variability estimated for the global land mass (Figure 3), primarily induced by climate fluctuations [24]. Particularly large between-year coefficients of variation in NPP are found for Africa's woodlands, savannas, and grasslands, according to one model incorporating satellite measurements of vegetation activity [25,26].

Africa plays a global role in C emissions through land use and fire (Table 1), though lack of information from the limited number of studies on the continent [e.g. [27-31]] restricts confidence in their magnitudes. Deforestation is the largest term in current assessments of tropical land use emissions [32], with Africa contributing 25% to 35% of total tropical land clearing from deforestation, and as much as 0.37 Pg C y^-1^, in the last decades [32,33]. Carbon losses through deforestation tend to be 'permanent' in Africa, as afforestation and reforestation rates are modest, at less than 5% of annual deforestation [32]. The associated net release of carbon from land use in sub-Saharan Africa is estimated to be 0.4 Pg C y^-1^, or 20% of the tropical total, nearly all attributed to deforestation [32]. Annual net C emissions from conversion to agriculture and cultivation practices alone are estimated [24] to be about 0.8 Pg C y^-1 ^for tropical land masses, but only 0.1 Pg C y^-1 ^from Africa [24,31], where shifting cultivation is prevalent [31].

Lack of information prohibits even the best land use change C emissions assessments from including all of the terms anticipated to be important for Africa. Pastoralism, shifting cultivation, and domestic wood harvest are widespread across the continent, but are often assumed to be inconsequential or are not considered [e.g. [32]], such that land use and land use change emissions from Africa are likely to be underestimated. Recent work [31] explicitly simulates aspects of these practices, though still focuses exclusively on forest and cropland conversions, missing land use change C emissions in Africa's vast savannas and grasslands which are home to much of the continent's livestock and the center of Africa's cereal and grain production. Furthermore, net C fluxes associated with changes in land use practices but not involving land conversion, such as management of tillage, slash, crop residues, and crop rotation, are refinements currently missing from continental scale land use change assessments. Finally, much of Africa, particularly in the semi-arid regions, is vulnerable to degradation, that may be the result of periodic drought or caused by agricultural and pastoral activities, releasing presumably large but unknown amounts of CO_2 _from cleared and dead vegetation [34] as well as possibly triggering strong biophysical feedbacks to the climate system [35] that may accelerate warming and prolong droughts [36-38].

Fire and land use emissions of carbon are entwined, especially in the humid and subhumid forest areas where fire is a primary tool for land transformation. Fire emissions associated with deforestation, shifting cultivation, burning of agricultural residues, and fuelwood may be as large as 2 Pg C y^-1 ^globally and 0.4 Pg C y^-1 ^for Africa, each of similar magnitude to estimates of total land use-related C emissions from those regions (Table 1). Consequently, estimates of land use change and deforestation C emissions already include, at least in theory, the associated fire emissions. New methods to estimate fire emissions using satellite sensors and atmospheric carbon monoxide measurements [39,40] will improve our ability to diagnose C emissions in fires.

Fire is also a common dry season occurrence in the seasonal savannas that encircle the humid forest zone. Carbon emissions in savanna fires represent a much shorter-term C loss than forest fires, since the main fuel is dead herbaceous vegetation, representing just one or two years of growth [27,41]. Thus savanna fires may only lead to faster cycling of biomass carbon rather than a net emission. Even if carbon emissions from savanna fires are roughly balanced over the long-term by growth in subsequent years, fires provide intense and localized injections of carbon into the atmosphere potentially shifting the seasonal or interannual distribution of CO_2 _releases [27,41]. Given the large magnitude of these fluxes in Africa, even fairly small (e.g. 20%) variation in year to year total fluxes could translate into annual variation in pyrogenic fluxes of 300 Tg of C or more. Correspondingly, recent results suggest that biomass burning is the largest source of interannual variability in land-atmosphere carbon fluxes [42].

Unlike respiration, fires return carbon to the atmosphere as a wide range of compounds, some of which are chemically or radiatively active (e.g. methane, carbon monoxide and aerosols), or are precursors to radiatively active gases (e.g. ozone precursors). Methane and other hydrocarbons, carbon monoxide, and black carbon releases in Africa are almost entirely of pyrogenic origin, and are thus included in the biomass burning term (Table 1) [27,28,41]. Methane consumption in upland soils is small, and available estimates of methane release from African wetlands suggest that they are globally insignificant [43]. However, given that there is no reliable map of wetland extent in Africa, and virtually no direct emission estimates, the true size of this flux is unknown. Recent work suggests the possibility of a large methane source of unknown magnitude from living plants [44,45]. Emissions of volatile organic compounds (VOCs) such as isoprene and monoterpenes have been studied in some detail in southern and central Africa and are estimated to return as much as 0.08 Pg C y^-1 ^to the atmosphere [46]. At the scale of the continent, industrial emissions of carbon dioxide, carbon monoxide and hydrocarbons from Africa are small, but can be locally very significant in the industrial areas of South Africa, the oilfields of the Gulf of Guinea, Angola, and Libya, and around major cities elsewhere in Africa.

The export of dissolved organic and inorganic carbon (DOC and DIC) in river water discharged to oceans is, by and large, offset by DOC and DIC delivered in precipitation (Table 1). Africa is also a minor net global source of biomass carbon through international exchange, mainly from export of wood products [47].

## What the Atmosphere Sees

**Figure 4:**
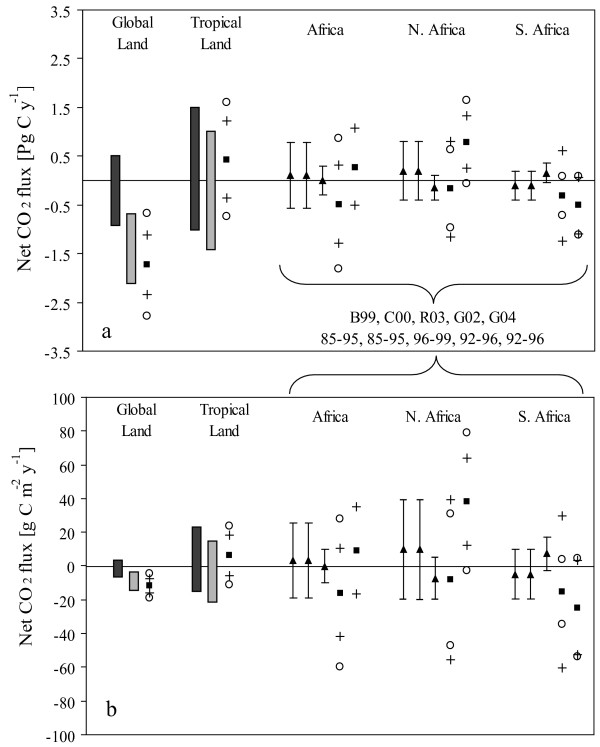
Terrestrial carbon source and sink estimates for Africa, tropical and global lands. (a) Net carbon dioxide flux totals, and (b) net carbon dioxide flux per unit area. Positive values indicate a surface source. Boxes show the range of +/- 1 standard deviation from the IPCC report [6] for global and tropical land during the 1980s (dark) and 1990s (light), whereas symbols report results from inverse analyses cited in Appendix I. Triangles and error bars indicate mean flux estimates from individual inversion studies and associated posterior uncertainties. Squares indicate the average, and pluses indicate the standard deviation, of mean flux estimates from a group of inverse solutions. Circles indicate the average uncertainty estimates among the group of inverse solutions. Atmospheric inversion results for Africa are taken from Bousquet [18] (B99), Ciais [107] (C00), Rödenbeck [51] (R03), Gurney [17] (G02), and Gurney [19] (G04), with years spanned in each analysis shown below literature source abbreviations of (a).

Atmospheric mixing ratios and isotopic compositions measured around the globe [48] can be used to estimate terrestrial and oceanic carbon sources and sinks by inversion with atmospheric transport models [18,49-51]. Inverse solutions for Africa are poorly constrained due to the lack of tropical, especially African, observations (Figure 4) [17,18]. This contributes to larger uncertainty ranges around net CO_2 _flux estimates for Africa than for global or tropical land areas, in general. Taken together, inversion results demonstrate that Africa's net role in global carbon cycling is highly uncertain. Furthermore, lack of data causes the inverse solution for southern Africa to trade off with solutions for South America and the southern oceans, such that results can vary widely between regions with no net change in overall source/sink strength [e.g. [17,19]]. Solving this problem will require the addition of precise, long-term observations of carbon dioxide in the tropics, located such that they help resolve the longitudinal differences among the southern hemisphere regions [52-54]. Tropical atmospheric dynamics present an additional challenge [55], and a source of uncertainty that is not represented in Figure 4, because deep convection is both poorly represented in transport models and poorly sampled, introducing non-negligible biases in atmospheric inversions.

Recognizing large uncertainties in the inverse solutions, inverse studies to date suggest that Africa as a whole is approximately carbon neutral on an annual to long-term basis. This is so despite significant carbon emissions related to land use change and burning, implying that net plant growth and corresponding sequestration of carbon in vegetation and soils is sufficiently large across the continent to offset the loss terms. If inverse solutions correctly estimate a carbon neutral Africa and assuming a neutral biosphere with a background balance between net primary production and heterotrophic respiration plus natural fires, the remaining biotic uptake or sequestration can be estimated as roughly offsetting Africa's land use (0.4 Pg C y^-1^) plus fossil fuel (0.2 Pg C yr-1) sources, still noting the large uncertainties.

**Figure 5:**
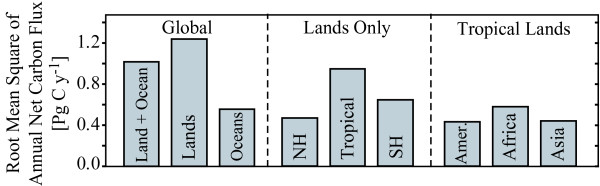
Root mean square of annual net carbon flux obtained from time-dependent inverse solutions [20] for the period 1990 – 2001 and for regions based on TransCom [20, 108]. NH (Northern Hemisphere land) includes temperate and boreal Asia, temperate and boreal North America, and Europe, SH (Southern Hemisphere land) includes temperate South America, Australia and New Zealand, Tropical includes tropical America, Africa and tropical Asia, and Amer. abbreviates America.

Despite a near-zero balance, recent time-dependent inverse solutions attribute much of the interannual variability (IAV) in global carbon sources/sinks to the African continent [20,42,51,56]. Estimates of regional IAV are less sensitive to transport and station-selection than seasonal and long-term mean fluxes [20]. Global solutions for the IAV of carbon sources/sinks [20] robustly indicate the strong influence of global lands, particularly those in the tropics, with approximately equal contributions by lands of tropical Asia, Africa, and southern and tropical America (each about 0.5 Pg C y^-1^) (Figure 5). However, temporal source/sink dynamics are still poorly constrained among tropical regions, especially those of Africa and America [20].

Taken together, large temporal variability of carbon sources and sinks may be Africa's most significant contribution to the global carbon cycle. This is consistent with results from ecosystem models [22-24], which indicate that high interannual variability in rainfall throughout the Sudano-Sahelian and southern African regions [57,58], partly associated with the North Atlantic Oscillation, El Niño Southern Oscillation, and South Pacific circulation [59,60], introduce pronounced multi-year fluctuations in surface-atmosphere C exchanges, which, in turn, appear in atmospheric CO_2 _concentrations [51]. Inter-annual variability in NPP then translate to variability in fire emissions with a lag of several months to a year. Such departures from long-term average biosphere exchange [51] and fire emissions [42,51] may both be as large as the net exchanges themselves.

## The Future of Carbon Cycle Research in Africa

Given the need to better understand the spatial and temporal dimensions of the global carbon cycle for prediction and management of future atmospheric CO2 concentrations, a number of research priorities for Africa emerge from this review. Of primary importance is the need for continent-wide observations that support both bottom-up and top-down methods of estimating carbon sources and sinks. Continued and new investment in collection and synthesis of carbon cycle information (measurements of carbon stocks and fluxes within and between the major pools) is needed to advance process-level characterization of seasonal and interannual variations in source/sink strengths. Such data will help to improve the way biophysical and biogeochemical models represent African ecosystems so that they capture the full suite of uniquely African features such as the continent's seasonal fire cycles, pastoralism, fuelwood harvest, cereal/grain production, dryland degradation, and the productivity and isotopic signatures of its extensive C4 vegetation. In particular, carbon flux observations wherever existing need to be used in model development and testing to appropriately represent the sensitivity of production and respiration processes to climate fluctuations. New collaborative research programs and networks are emerging in Africa to address some of the gaps through expanded site-based and regional field measurements and model-based analyses (including, amongst many national and regional activities, the growing network of  eddy covariance sites (the "Afriflux" network), the African Monsoon Multidisciplinary   Analysis (AMMA), the South African Ecological Observation Network (SAEON), and the Environmental Long-Term Observatories of southern Africa (ELTOSA)).

Support for inventory and monitoring of soil and vegetation carbon stocks by forest and agricultural research stations, long-established in most African countries [61], is critical. The associated national resource inventories [62] provide information invaluable for assessing regionally-specific ecosystem responses to natural and human disturbances and for anchoring regional-scale estimates of land use related carbon sources and sinks. Synthesis of country-level data is of great importance to provide local and regional scale data to underpin regional- and global-scale carbon cycle assessments. These will complement developments in satellite remote sensing of vegetation biomass using, for example, passive and active optical and radar approaches [63,64].

A well-located atmospheric sampling network in Africa is also needed to better constrain inversion estimates of regional carbon sources and sinks and their temporal variability both in Africa and globally. However, improved constraint relies not only on new observations but also improvement of modeled transport and inverse estimation techniques. New transport schemes are needed to represent deep tropical convection, while new data assimilation and computational techniques promise to better resolve the African signal in global atmospheric carbon dynamics by incorporating diurnal variation in surface fluxes, multiple atmospheric tracers, and prior estimates of fire emissions. With regard to additional carbon cycle tracers, Africa is unique in having vast coverage of C4 vegetation [65], associated with a prevalence of semi-arid and hot environments. Seasonal production, respiration, and burning of C4 vegetation alter the carbon stable isotope (^13^C:^12^C) composition of the atmosphere because C4 plants discriminate against the heavier isotope less than C3 plants. This imprint provides a tracer for diagnosing Africa's role in global carbon stocks and fluxes [40], presenting a potential opportunity for separation of moist tropical forest exchange from that of the savanna regions. Furthermore, since oceanic and C4 plant discrimination are similar, information on the C4 terrestrial exchange is critical for separation of terrestrial from oceanic fluxes.

An orbiting space-based total column carbon observatory covering the entire globe is anticipated within the next decade [66], but it will still require near-surface and vertical profile measurements of CO_2 _for calibrating, validating, interpolating and interpreting satellite-derived observations. Satellite-based assessments of local to regional vegetation change from land use practices [e.g. [67]] should be further explored for continental-scale assessment. These and other data could be used to develop land use/land cover transition models that represent Africa's unique human-vegetation-climate settings. Such comprehensive investigations into regionally-specific ecosystem responses to land use in Africa offer needed detail for representing the complex dynamics associated with human-induced disturbances and land use management.

## Africa and the Climate Change Context

Recent International Panel on Climate Change (IPCC) assessments show that industrialized nations are imposing a heavy burden of climate change on the global environment through emissions of carbon dioxide (CO_2_) and other greenhouse gases, largely from the burning of fossil fuels [6]. Non-industrialized countries currently contribute little to these emissions, but are vulnerable to climate change and will therefore be forced to take potentially costly measures to adapt.

Nearly all African countries are signatories to the UN Framework Convention on Climate Change (UNFCCC) and, being non-Annex 1 countries, there is no cap on their greenhouse gas emissions in the first Kyoto Protocol commitment period. Still, parties in Africa can participate in the Clean Development Mechanism (CDM) of the Kyoto Protocol, under which developed countries that have accepted emission caps are authorized to implement projects that reduce emissions or sequester carbon in developing countries. The resulting certified emissions reductions can then be used to meet a fraction of Annex 1 emission targets. This mechanism provides opportunities for less developed countries to leap-frog to clean industries using foreign investment and technology.

The predominantly agricultural nations of Africa are poorly-positioned to benefit financially and technologically from emission mitigation trading schemes, insofar as these mechanisms focus mainly on industrial emissions reductions, which are more easily verified. However, the scope for carbon sequestration through management of land in developing countries is large, and CDM provisions for land use based carbon emission reductions might provide rapid, medium-term sequestration at relatively low cost. Uncertainties surrounding the quantification and verification of carbon sequestration through changes in land management have thus far prevented large-scale investment in this strategy. This situation could change with improved understanding of carbon cycle dynamics in terrestrial ecosystems and suitable verification schemes, enabling many African nations to more easily participate in global efforts to slow the rate of increase of atmospheric CO_2_, as well as benefit from the financial and technological transfers.

Carbon sequestration through reforestation of lands deforested prior to 1990 appears to be one of the most readily available opportunities for a number of African countries. Fire management presents another prospective opportunity for mitigation, but reducing fire occurrence has proven difficult in the past [e.g. [68]], and such programs would need to be wary of unintentional loss of biodiversity from fire-adapted biota. Climate change mitigation through land management could also impart unintended environmental and social costs that affect the most vulnerable sectors of society, for example from converting lands in subsistence farming to large scale carbon plantations, or by restriction of fuel wood harvest for domestic uses. Such programs therefore require careful evaluation of the potential costs and benefits, particularly for already marginalized populations.

## A Global Outlook

With as much as 40% of the world's fire emissions, about 20% of global net primary production and heterotrophic respiration, at least 20% of global land use emissions, and a major source of interannual variability in global net carbon exchange, African carbon dynamics are of global significance. The continent's vast carbon stocks seem to be highly vulnerable to climate change, evidenced by strong sensitivity of net ecosystem productivity and fire emissions to climate fluctuations. Being highly variable and insufficiently studied, there is a need for continued and enhanced observations of Africa's carbon stocks, fluxes, and atmospheric concentrations to enable more precise assessments of Africa's carbon cycle, and its sensitivity to natural and anthropogenic pressures and future climate.

In years ahead, Africa's land use pressures will undoubtedly increase and climate changes are anticipated to intensify drought cycles and make much of Africa warmer and dryer [69]. Furthermore, increasing exploitation of forest resource in the moist tropics is anticipated with economic development and investment in logging infrastructure. Such changes will likely release CO_2 _to the atmosphere as well as increase the magnitude of interannual variation in Africa's C fluxes by increasing Africa's biomass burning emissions and reducing the continent's net ecosystem productivity. If realized, these trends would have enormously important implications for global carbon dynamics and biospheric feedbacks to the climate system.

## Competing interests

The author(s) declare that they have no competing interests.

## Authors' contributions

All authors participated in detailed discussions that led to this review paper. CAW compiled and analyzed the data and drafted the manuscript. NPH originally conceived the paper and contributed to data analyses, interpretation, drafting and editing the manuscript. JCN, RJS, JAB and ASD provided intellectual input on available data and previous analyses, and on the synthesis, presentation and interpretation needed for this review. DFB made data available from a global time-dependent inverse analysis of CO_2 _concentrations contributing to Figure 5. All of the authors read, edited, and approved the final manuscript.

## Appendix 1. Methods

Table 1 contains statistics of a sample of independent mean estimates for each term, presenting arithmetic means of reported values, their maxima and minima, and standard deviations. When a standard deviation around a mean estimate was reported in one of the many sources, we attempted to incorporate some of this uncertainty in the tabular values of Table 1 by including the mean, mean plus standard deviation, and mean minus standard deviation all as independent estimates contributing to the sample.

Annual precipitation C flux to Africa was estimated from the sum of estimates for dissolved organic and inorganic (DOC and DIC) carbon fluxes from precipitation following the approach in Kempe [70]. For DOC, the flux was calculated as the product of annual precipitation water flux with the maximum or minimum observed continental rainwater DOC reported in Willey et al. [71], where precipitation delivered to Africa was estimated from an FAO rainfall product [72]. Similarly, the DIC flux was calculated as the product of annual African precipitation with a) continental rainwater DIC at a pH of 7.4 and 10°C as in Willey et al. [71], and b) its product with the mean CO_2 _content of precipitation reported in Miotke [73].

Africa's annual riverine C discharge to oceans was calculated from the sum of riverine DOC and DIC flux estimates also as in Kempe [70]. For DIC the flux was calculated as the product of Africa's riverine discharge [74,75] with DIC content of Africa's river water [74]. For DOC, we used the global ratio of DOC to DIC in river water [74] to estimate DOC content of Africa's river water, which was then multiplied by river water discharge.

When not directly reported, carbon emissions from human-managed fires were estimated by converting biomass burned into carbon emissions based on a common [e.g. [27,28,76]] assumption of biomass to carbon emissions ratio of ~0.45.
